# TLR9 stimulation of B-cells induces transcription of p53 and prevents spontaneous and irradiation-induced cell death independent of DNA damage responses. Implications for Common variable immunodeficiency

**DOI:** 10.1371/journal.pone.0185708

**Published:** 2017-10-03

**Authors:** Kristine Lillebø Holm, Randi Gussgard Syljuåsen, Grete Hasvold, Lene Alsøe, Hilde Nilsen, Kristina Ivanauskiene, Philippe Collas, Sergey Shaposhnikov, Andrew Collins, Randi Larsen Indrevær, Pål Aukrust, Børre Fevang, Heidi Kiil Blomhoff

**Affiliations:** 1 Department of Molecular Medicine, Institute of Basic Medical Sciences, University of Oslo, Oslo, Norway; 2 Department of Radiation Biology, Institute for Cancer Research, Oslo University Hospital, Radiumhospitalet, Oslo, Norway; 3 Department of Clinical Molecular Biology, University of Oslo and Akershus University Hospital, Lørenskog, Norway; 4 Comet Biotech AS, Norgenotech AS, Oslo, Norway; 5 Department of Nutrition, Institute of Basic Medical Sciences, University of Oslo, Oslo, Norway; 6 Section of Clinical Immunology and Infectious Diseases, Oslo University Hospital, Rikshospitalet, Oslo, Norway; 7 Research Institute of Internal Medicine, Oslo University Hospital, Rikshospitalet, Oslo, Norway; ENEA Centro Ricerche Casaccia, ITALY

## Abstract

In the present study, we address the important issue of whether B-cells protected from irradiation-induced cell death, may survive with elevated levels of DNA damage. If so, such cells would be at higher risk of gaining mutations and undergoing malignant transformation. We show that stimulation of B-cells with the TLR9 ligands CpG-oligodeoxynucleotides (CpG-ODN) prevents spontaneous and irradiation-induced death of normal peripheral blood B-cells, and of B-cells from patients diagnosed with Common variable immunodeficiency (CVID). The TLR9-mediated survival is enhanced by the vitamin A metabolite retinoic acid (RA). Importantly, neither stimulation of B-cells via TLR9 alone or with RA increases irradiation-induced DNA strand breaks and DNA damage responses such as activation of ATM and DNA-PKcs. We prove that elevated levels of γH2AX imposed by irradiation of stimulated B-cells is not due to induction of DNA double strand breaks, but merely reflects increased levels of total H2AX upon stimulation. Interestingly however, we unexpectedly find that TLR9 stimulation of B-cells induces low amounts of inactive p53, explained by transcriptional induction of *TP53*. Taken together, we show that enhanced survival of irradiated B-cells is not accompanied by elevated levels of DNA damage. Our results imply that TLR9-mediated activation of B-cells not only promotes cell survival, but may via p53 provide cells with a barrier against harmful consequences of enhanced activation and proliferation. As CVID-derived B-cells are more radiosensitive and prone to undergo apoptosis than normal B-cells, our data support treatment of CVID patients with CpG-ODN and RA.

## Introduction

The integrity of the human genome is constantly threatened by exposure to genotoxic stress from both endogenous and exogenous sources [1;2]. Endogenous sources alone, such as those from metabolic processes, account for more than 10,000 lesions per cell per day [3]. DNA damage, if not repaired, play major roles in carcinogenesis, aging and related diseases such as metabolic and cardiovascular disorders [4]. Therefore, use of DNA-damaging agents like irradiation in clinical settings raises the awareness of possible side effects, such as immune dysregulation and development of secondary cancers [5;6]. Lymphocytes, and particularly B-cells, are regarded as highly sensitive to irradiation, as they easily respond by undergoing apoptosis [7]. We have previously shown that stimulating B-cells with the vitamin A metabolite retinoic acid (RA) in combination with ligands crosslinking TLR9 protects B-cells against both spontaneous and irradiation-induced apoptosis [8].

Protection of B-cells against irradiation is particularly relevant for patients diagnosed with primary immunodeficiencies affecting B-cells functions, such as Common variable immunodeficiency (CVID). Although malfunctioning T-cells and monocytes/macrophages may contribute to the immunodeficiency, the hypogammaglobulinemia characteristic of CVID patients is most often linked to dysfunctional B-cells [9]. It has been reported that CVID patients have increased levels of reactive oxygen species (ROS) and disturbed regulation of glutathione metabolism suggesting enhanced oxidative stress [10;11]. Oxidative stress might predispose to increased DNA damage, and it has been shown that B-cells from CVID patients display higher levels of spontaneous and DNA damage-induced apoptosis [12;13]. This is critical for such patients, as they are frequently exposed to diagnostic X-rays due to serious recurrent infections and other complications [14]. The increased sensitivity to apoptosis may contribute to the reduced numbers of viable B-cells in these patients, and thus maintaining the immune deficiency [15]. Furthermore, the documented increased risk of CVID patients to develop malignancies such as lymphomas [14], has been ascribed to a possible inborn cellular radiosensitivity due to increased chromosomal instability [16]. Whereas the molecular causes of CVID are largely unknown in most of the patients, several studies point to defects in TLR9-mediated responses [17;18]. Interestingly, it has also been reported that CVID patients frequently suffer from vitamin A deficiency [19;20]. The disturbances in TLR9 signaling and vitamin A metabolism could potentially contribute to an increased sensitivity to irradiation in B-cells from these patients.

Irradiation results in a wide range of DNA lesions in exposed cells [21;22], such as base damage, single strand breaks (SSBs) and double strand breaks (DSBs). DSBs are the most deleterious type of damage, where in certain cases as little as one DSB is sufficient to induce apoptosis [23]. To preserve genomic integrity, mechanisms have evolved in eukaryotes to deal with such harmful lesions–collectively termed as the DNA damage response (DDR) [1;2]. The DDR consist of a hierarchy of proteins that through the action of sensors, transducers and effectors determine the fate of cells harboring DNA damage. In response to DSBs, for instance caused by irradiation, the kinases ataxia telangiectasia mutated (ATM) and DNA-dependent protein kinase catalytic subunit (DNA-PKcs) are recruited to the site of damage and activated [24;25]. Activated kinases will in turn phosphorylate downstream substrates such as the histone variant H2AX at S139 to form γH2AX [26]. γH2AX plays a role in maintaining genomic integrity and is required for the localization of DNA repair proteins at the site of damage [27]. DDR pathways converge in the accumulation and activation of the tumor suppressor p53 [2;28]. As a key factor in the DDR, p53 acts as a transcription factor that upon its stabilization and activation regulates genes involved in cell cycle arrest, DNA repair, senescence and apoptosis [29].

Having established that RA in combination with TLR9 stimulation rescues normal B-cells from irradiation-induced apoptosis by increasing expression of the anti-apoptotic factor myeloid cell leukemia 1 (MCL1) [8], we were concerned about the possibility of cells surviving with elevated levels of DNA damage. If so, treatment with the TLR9-ligands CpG-oligodeoxynucleotides (CpG-ODN) alone or with RA would increase mutation load and possibly tumorigenesis in B cell from both healthy subjects and CVID patients. We show that TLR9 stimulation with or without RA protects both normal and CVID-derived B-cells from spontaneous and irradiation-induced cell death. Importantly, the enhanced survival is not accompanied by elevated levels of DNA strand breaks and DDR activation. However, an unexpected induction of p53 in TLR9/RA-stimulated B-cells suggest that the cells may be provided with a “fit for fight” protective barrier against potentially harmful consequences of enhanced B-cell activation and proliferation.

## Materials and methods

### Patients and healthy controls

Normal B-cells were isolated from buffy coats prepared from healthy blood donors admitted to the Blood Bank (Oslo University Hospital, Oslo, Norway). For studies on B-cells from CVID patients, blood samples (36 ml) from 7 CVID patients (2 males and 5 females, mean age 53.4 ±16.2 years) and 7 healthy controls (mean age 34.8 ±8.4 years) were included after informed written consent. Patient characteristics are presented in S1 Table. The patients were recruited from Section for Clinical Immunology and Infection Medicine at Oslo University Hospital, Rikshospitalet, between May and August 2016. CVID patients were diagnosed according to criteria from the International Union of Immunological Societies scientific committee [30;31]. Patients that were known to have very low levels of B-cells were excluded due to the numbers of B-cells required for these studies. All patients regularly received subcutaneous and/or intravenous immunoglobulin replacement therapy and did not suffer from acute infections at the time of blood collection. For patients on intravenous replacement therapy blood samples were collected immediately prior to Ig infusion. The healthy controls were recruited from the Department of Molecular Medicine, Institute of Basic Medical Sciences, University of Oslo. The studies on B-cells from healthy blood donors and CVID-derived B-cells were approved by the Regional Committee for Medical and Health Research Ethics in South-Eastern Norway (REK 2.2006.3062 and 2012/521, respectively). The investigation conforms to the principles outlined in the Declaration of Helsinki.

#### Reagents and antibodies

Modified CpG oligodeoxynucleotide phosphorothionate 2006 was purchased from Enzo Life Science (Farmingdale, NY, USA) and purified monoclonal mouse anti-CD180 (RP105) antibody (312902) was obtained from BioLegend (San Diego, CA, USA). All-*trans* retinoic acid and propidium iodide (PI) were from Sigma-Aldrich (St. Louis, MO, USA). Monoclonal mouse anti-phospho-H2AX (S139; 05–636) and polyclonal rabbit anti-H2AX (AB10022) antibodies used in flow cytometry were purchased from Merck Millipore (Billerica, MA, USA) and used at the final dilution 1:250 and 1:100, respectively. Secondary antibodies Alexa Fluor 488-conjugated polyclonal goat anti-mouse antibody (A21202) or anti-rabbit antibody (A21206) were obtained from Molecular Probes (Eugene, OR, USA) and were used at the final dilution 1:1000 and 1:500, respectively. For immunofluorescence analyses we used monoclonal mouse anti-phospho-H2AX antibody (S139; 05–636) at the final dilution 1:1500 and Alexa Fluor 488-conjugated polyclonal donkey anti-mouse antibody (715-545-150, Jackson Immunoresearch laboratories, West Grove, PA, USA) at the final dilution 1:200. FxCycleTM Far Red from Thermo Fisher Scientific (Waltman, MA, USA) was used as a DNA stain in flow cytometry analyses, and DAPI (Sigma-Aldrich) was used as a DNA stain in immunofluorescence analysis.

Antibodies used for immunoblotting: Antibodies for detecting calnexin (2433), phospho-p53 (S15; 9284) and phospho-ATM (S1981; 5883) were purchased from Cell Signaling (Danvers, MA, USA). All antibodies from Cell Signaling were polyconal rabbit antibodies and were used at the final dilution of 1:1000. Monoclonal mouse anti-p53 antibody (DO-1; sc-126) was obtained from Santa Cruz Biotechnology (Dallas, TX, USA) and used at final dilution 1:200, whereas monoclonal mouse anti-p21^Cip^ (554228) was purchased from BD Bioscience Pharmingen (Franklin Lakes, NJ, USA) and was used at the final concentration 1 μg/ml. The secondary polyclonal goat anti-mouse (170–6516) and goat anti-rabbit (170–6515) antibodies were purchased from Bio-Rad (Hercules, CA, USA) and used at the final dilution 1:5000. Precision Blue protein standard was obtained from Bio-Rad.

### B-cell isolation, culturing and radiation treatment

B-cells from buffy coats, CVID patients and healthy controls were isolated and cultivated in the same manner. Resting human B-cells were isolated from buffy coats or samples of whole blood by using anti-CD19 antibody-coated Dynabeads (Life Technologies, Carlsbad, CA, USA) and CD19 DETACHaBEADS (Life Technologies) as described [32]. The purity of the isolated B-cells was ≥98%. B-cells were cultured in RPMI 1640 (Lonza, Basel, Switzerland) containing 10% heat-inactivated fetal bovine serum (Sigma-Aldrich), 125 U/ml penicillin and 125 U/ml streptomycin (Sigma-Aldrich) at 37°C in a humidified incubator with 5% CO_2_. For all experiments freshly isolated CD19+ B-cells were cultured at 0.5 x 10^6^ cells/ml in various combinations of CpG-ODN (0.5 μg/ml) and RA (200 nM) for 24 hours before irradiation (10 Gy).

DNA damage was induced by irradiating cells using the Xstrahl RS320 radiation equipment (Xstrahl, Surrey, UK) at a rate of 3.9 Gy/min. Cells were irradiated at 10 Gy unless otherwise indicated.

### Determination of cell viability

Cell death was analyzed in the flow cytometry instrument FACS Calibur (BD Biosciences, San Jose, CA, USA). Cell viability was determined by PI-exclusion test, where short-term incubation of cells in PI results in selective labeling of dead cells. Cells were incubated in 20 μg/ml PI for 15 minutes prior to analysis, and 10 000 cells where analyzed from each sample. The percent PI-positive cells was calculated using CellQuest software (BD).

### Western blot analysis

B-cells were harvested and lysed in RIPA-buffer (50 mM Tris [pH 7.5], 150 mM NaCl, 1% NP-40, 0.1% SDS, 0.5 mM EDTA, 50 mM NaF, 10 mM β-glycerophosphate, 1 mM Na_3_VO_4_, 0.2 mM phenylmethylsulfonyl fluoride, 10 μg/ml leupeptin, and 0.5% aprotinin), and protein concentration was determined by the use of the Pierce BCA protein kit (Thermo Fisher Scientific). Equal amounts of proteins were loaded on and separated by SDS-PAGE. After transfer to an Immobilon–P membrane (Merck Millipore) proteins were detected by standard immunoblotting procedures, using the enhanced chemiluminescence detection system SuperSignal^®^ West Dura Extended Duration substrate (Thermo Fisher Scientific). Images were acquired with a Syngene ChemiGenious camera and presented using GeneSnap (Syngene, Cambridge, England). Signal intensity was calculated using GeneTool (Syngene).

### Multiparameter flow cytometry and immunofluorescence

For analysis of the DNA damage marker γH2AX by flow cytometry, cells were fixed with 70% ethanol and stained as described [33]. Barcoding of sets of four samples with Pacific Blue was used to eliminate variations in antibody staining between samples [33]. DNA was stained using FxCycleTM Far Red (200nM FxCycle and 0.1mg/ml RNase A). Analysis was performed on a LSRII flow cytometer (BD Biosciences, Franklin Lakes, NJ, USA) using FACS Diva and FlowJo software (Ashland, OR, USA). Individual samples were separated by gating based on the Pacific Blue signal, and median γH2AX levels were determined.

For analysis of γH2AX by immunofluorescence, B-cells were attached to microscope slides by cytospin centrifugation before undergoing fixation (3% paraformaldehyde) and permabilization (0.1% Triton X-100, 2% bovine serum albumin, 0.01% Tween 20). DNA was stained with 0.1 μg/mL DAPI and coverslips mounted in Mowiol 4–88 (Polysciences, Warrington, PA, USA). The induction of γH2AX was measured using Olympus IX71 upright microscope (Tokyo, Japan) fitted with the DeltaVision system (GE Healthcare Life Science, Pittsburgh, PA, USA) and the data were processed by ImageJ 1.42q.

### Comet assay analysis of DNA damage

Comet assay was performed essentially as described [34]. Briefly, B-cells were embedded in 1% agarose, irradiated (5 Gy) and either immediately lysed in cold lysis buffer (2.5 M NaCl, 0.1 M EDTA, 10 mM Tris, 1% Triton X-100, pH 10) or allowed for repair in culture media for indicated amount of time before lysis. Electrophoresis at 0.8 V/cm was carried out for 20 min in a cold room. After neutralization with PBS and staining with SYBRGold (Invitrogen, Carlsbad, CA, USA), slides were scored (50 nucleoids per gel) using the Comet Assay IV image analysis program (Perceptive Instruments, Bury St Edmunds, UK).

### Quantitative mRNA analysis

RNA was isolated from the cell pellets using the RNeasy Plus Mini Kit (Qiagen, Valencia, CA, USA) and subjected to reverse transcription by iScript cDNA Synthesis Kit (Bio-Rad, Hercules, CA, USA). RT-qPCR was performed using CFX96™ Real-Time PCR Detection system (Bio-Rad) and SsoFast™ EvaGreen® Supermix (Bio-Rad). Primers towards *TP53*, *ATM*, *DDB1*, *BRCA2*, *OGG1*, *UNG*, *MSH2*, *ERCC1*, *TBP* and *B2M* were obtained from Qiagen. The amounts of target mRNA related to reference genes (*TBP* and *B2M*) were quantified using the 2^-ΔCt^-method.

### Statistical analysis

Statistical analysis of the data was performed using IMB SPSS Statistics 24 (Armonk, NY, USA). The Mann Whitney *U* test was used to compare different groups, and the Wilcoxon signed-rank test or paired *t*-test was used to compare different stimuli within groups. *P* values < 0.05 were considered significant.

## Results

### RA enhances TLR9-mediated survival of CVID-derived B-cells

**Fig 1:**
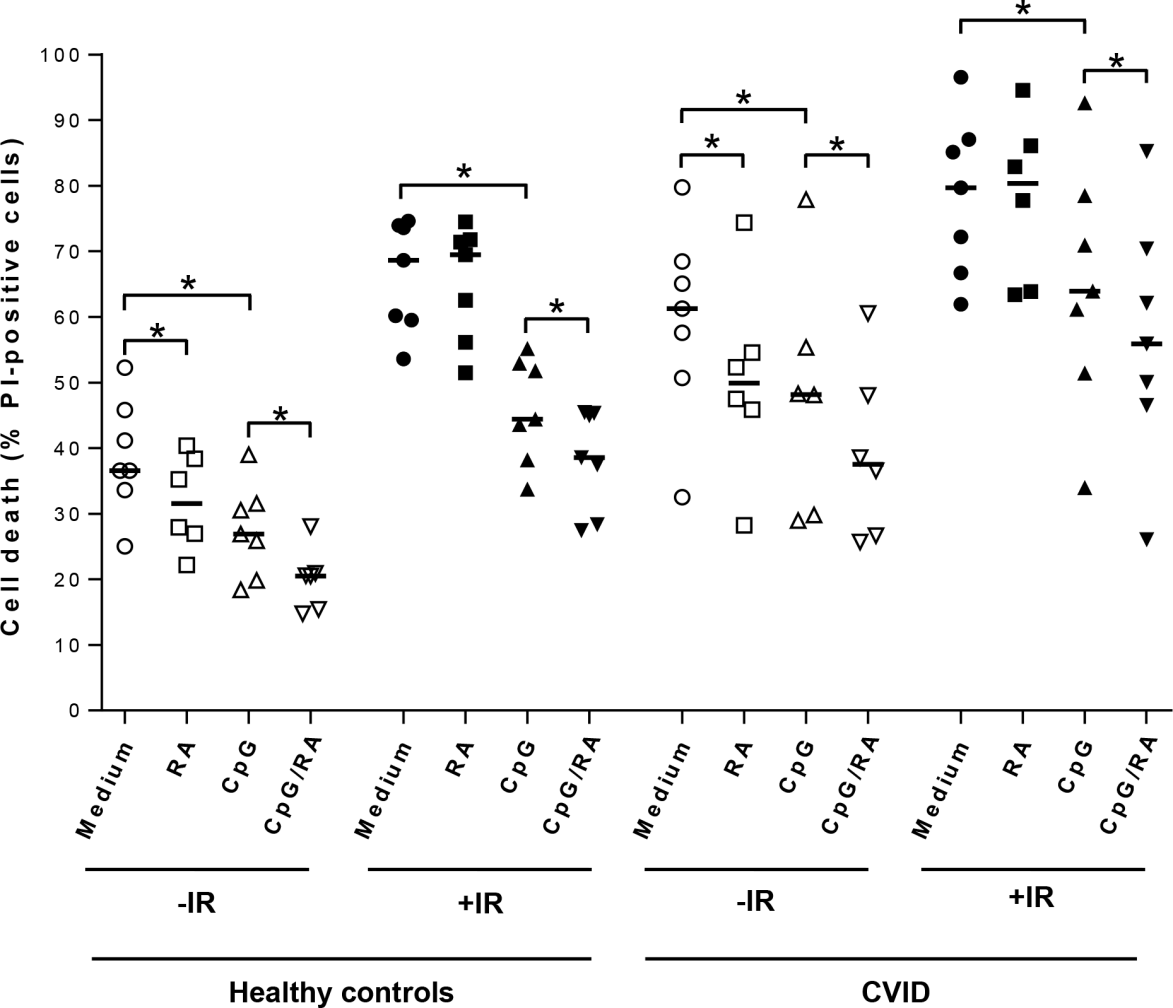
RA reduces spontaneous and irradiation-induced apoptosis of normal and CVID-derived B-cells stimulated via TLR9. Normal and CVID-derived B-cells were treated with CpG-ODN (0.5 μg/ml) in the presence or absence of RA (200 nM) for 24 hours prior to irradiation (IR; 10 Gy). After additional 24 hours, cell death was measured as the percentage of PI-positive cells. Each symbol represents the data from an individual patient or healthy control, and the horizontal lines represent median values, (n = 7, *p < 0.05, Wilcoxon signed rank test).

We aimed to explore the protective role of RA in combination with CpG-ODN on spontaneous and irradiation-induced cell death in B-cells from CVID patients. To this end, freshly isolated B-cells from CVID patients and healthy controls were stimulated with CpG-ODN in the presence or absence of RA for 24 hours prior to irradiation. Cell death was measured by staining with PI after additional 24 hours. We observed enhanced spontaneous and irradiation-induced cell death in CVID-derived B-cells as compared to B-cells from healthy controls (Fig 1). As also shown in Fig 1, CpG-ODN prevented the death of both normal and CVID-derived B-cells. However, we found that the CVID derived B-cells were significantly less protected from irradiation-induced cell death by CpG-ODN as compared to normal B-cells (20% versus 31% protection, *p* < 0.05; Mann-Whitney *U* test). Interestingly, the potentiating effect of RA on TLR9-mediated protection was comparable in CVID-derived B-cells and B-cells from healthy controls. Notably, RA in combination with CpG-ODN reduced irradiation-induced death of CVID-derived B-cells by 30% (p = 0.01; Wilcoxon signed rank test).

### TLR9 stimulation augments IR-mediated p53 levels

Survival of cells exposed to irradiation could theoretically be the result of reduced DNA damage or increased DNA repair, and if so be beneficial for an organism. However, agents increasing survival of cells exposed to irradiation or other DNA damaging mediators could also potentially increase the risk of promoting survival of cells with unrepaired DNA, thereby facilitating genomic instability. Thus, we explored the DDR in irradiated B-cells stimulated via TLR9 with or without RA. Of note, due to limited numbers of B-cells obtained from blood samples of CVID patients, these experiments could only be performed on B-cells from healthy blood donors.

**Fig 2:**
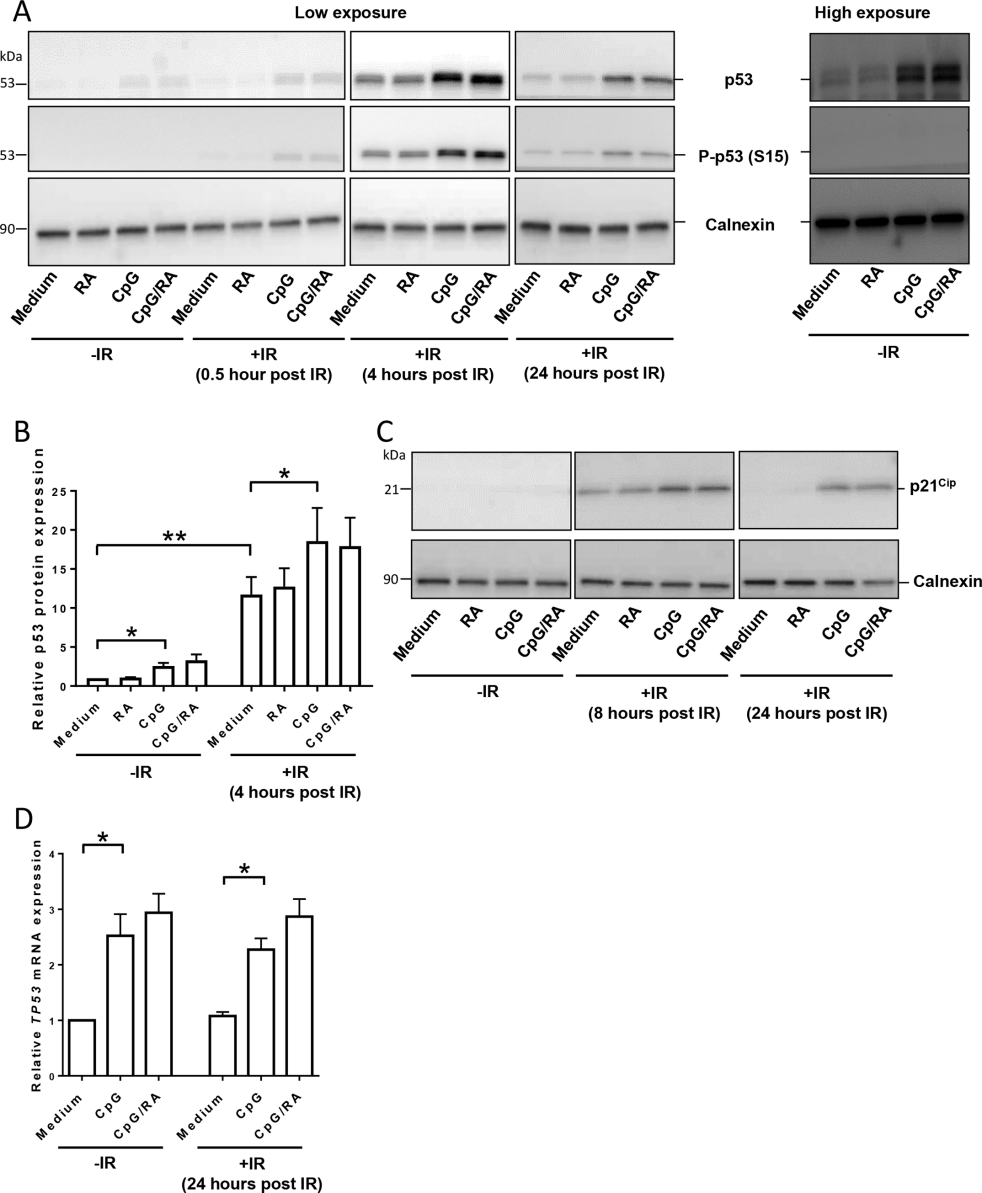
TLR9 stimulation induces p53 expression. **(A)** Normal B-cells were treated with CpG-ODN (0.5 μg/ml) in the presence or absence of RA (200 nM) for 24 hours prior to irradiation (IR; 10 Gy). After indicated time points B-cells were collected and subjected to western blot analysis with antibodies recognizing calnexin (as loading control), p53, or p53 phosphorylated at serine 15 (S15). The right panel shows a more exposed version of the blot depicting induction of p53 in non-irradiated cells. One representative experiment of three is shown. Original uncropped blots are presented in S5 Fig. **(B)** Densitometric analysis of the p53 expression (based on low exposure) at 4 hours after irradiation was normalized to calnexin. The results are presented as histograms of mean values ±SEM (n = 6, *p < 0.05, **p < 0.01, paired t-test). **(C)** B-cells were treated as described in (A), and lysates were subjected to western blot analysis with antibodies recognizing calnexin (as loading control) and p21^Cip^. One representative experiment of three is shown. Original uncropped blots are presented in S6 Fig. **(D)** B-cells were stimulated as described in (A), and the cells were harvested 24 hours after irradiation. The mRNA level of *TP53* was quantified by RT-qPCR. The amounts of *TP53* mRNA related to reference genes (*TBP* and *B2M*) were quantified using the 2^-ΔCt^-method. The results are presented as histograms of mean values ±SEM (n = 4 *p < 0.05, paired t-test).

P53 is regarded as a key factor in the DDR [35;36] and is regulated at both transcriptional, post-transcriptional and post-translational levels [37]. Under normal conditions, the level of p53 is low due to its short half-life. Upon DNA damage, the level of p53 increases within minutes mainly due to post-translational events such as acetylation and phosphorylation of p53 and its regulatory proteins [38]. P53 phosphorylation, notably at S15, is also required for its activation as a transcription factor [39].We found that CpG-ODN enhanced the levels of p53 induced by irradiation, with the highest induction at 4 hours post irradiation (Fig 2A and 2B). Of note, RA did not have any additional effect on the induction of p53 (Fig 2A and 2B). As expected, phosphorylation of p53 at S15 was only detected in the irradiated cells (Fig 2A). The levels of phosphorylated p53 followed that of total p53 in irradiated cells, and hence TLR9 stimulation also enhanced the irradiation-induced levels of phosphorylated p53. The activity of p53 was verified by the irradiation-induced expression of the p53-target gene p21^Cip^_,_ and again TLR9 stimulation augmented the effect of irradiation without additional effects of RA (Fig 2C).

We next assessed the effect of CpG-ODN in non-irradiated cells. We revealed that B-cells stimulated with CpG-ODN for 24 hours showed enhanced levels of p53, with again, no additional effect of RA (Fig 2A and 2B). Moreover, TLR9 stimulation did not affect the levels of phosphorylated p53 or p53 activity in non-irradiated B-cells (Fig 2A and 2C). The TLR9-mediated induction of p53 could be explained either by CpG-ODN somehow inducing DNA damage, or it could be a result of CpG-ODN simply increasing transcription of p53. Indeed, CpG-ODN alone upregulated *TP53* transcripts in non-irradiated cells already after 24 hours (S1 Fig) and more pronounced after 48 hours (Fig 2D), with no additional effect of RA or irradiation treatment.

### TLR9 stimulation does not enhance irradiation-mediated activation of the DDR upstream of p53

**Fig 3:**
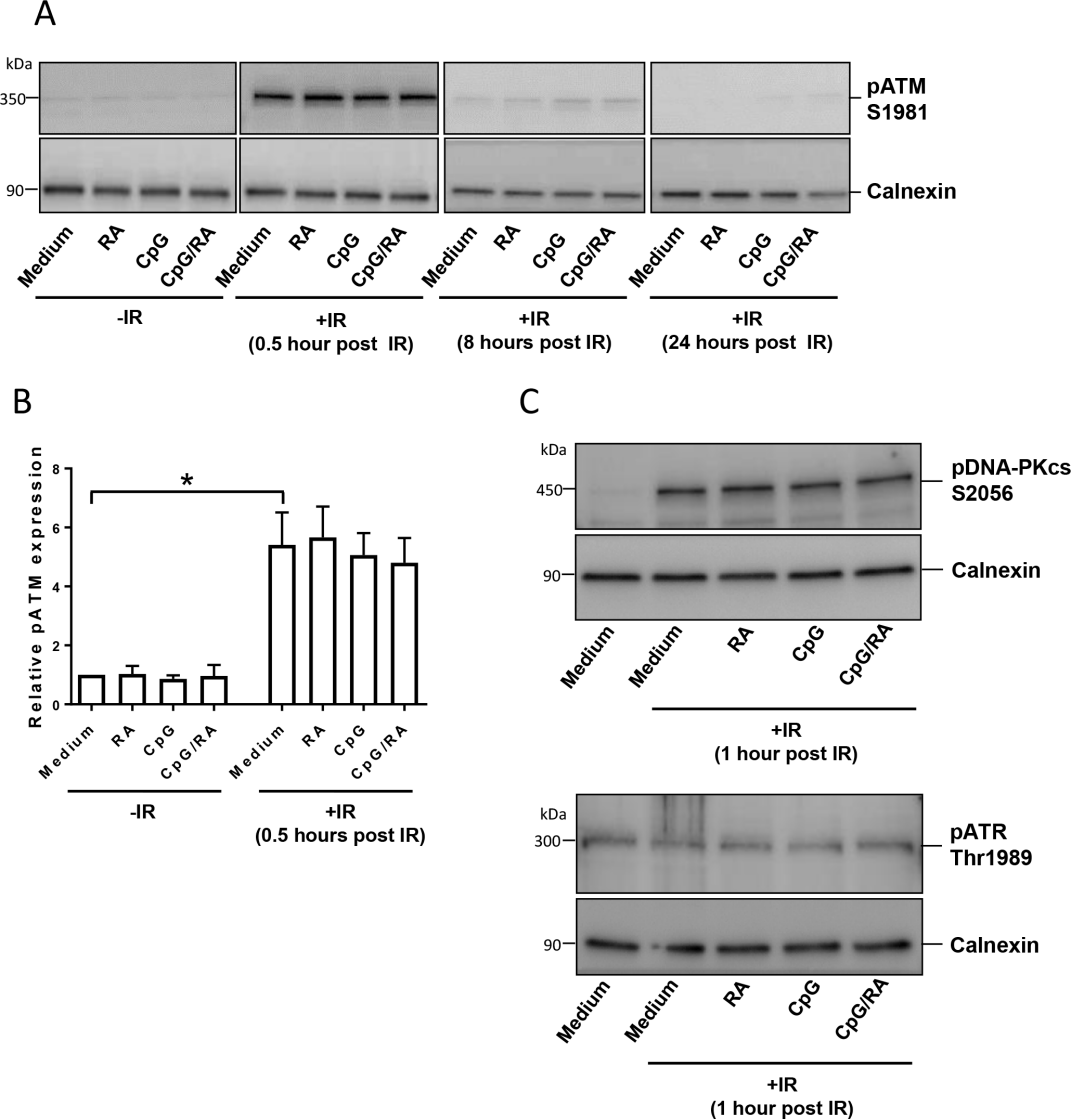
TLR9 stimulation does not activate DDRs upstream of p53. **(A)** B-cells were treated with CpG-ODN (0.5 μg/ml) in the presence or absence of RA (200 nM) for 24 hours prior to irradiation (IR; 10 Gy). After indicated time points, B-cells were collected and subjected to western blot analysis with antibodies recognizing calnexin (as loading control) and phosphorylated ATM (S1981). One representative experiment of three is shown. Original uncropped blots are presented in S7 Fig. **(B)** Densitometric analysis of pATM expression at 0.5 hours after irradiation was normalized to calnexin. The results are presented as histograms of mean values ±SEM (n = 5, *p < 0.05, paired t-test). **(C)** B-cells were treated as indicated in (A), and the B-cells were harvested one hour after irradiation and subjected to western blot analysis with antibodies recognizing calnexin (as loading control), pDNA-PKcs (S2056, upper panel) and pATR (Thr1989, lower panel). One representative experiment of three is shown. Original uncropped blots are presented in S8 Fig.

In light of the induced expression of p53, it was important to verify that TLR9 stimulation did not promote activation of the DDR upstream of p53. An early step in the DDR is the phosphorylation and activation of PI3K-family kinases such as ATM, ataxia telangiectasia and rad3-related protein (ATR) and DNA-PKcs [24;25]. We found that irradiation induced phosphorylation of ATM and DNA-PKcs (Fig 3A, 3B and 3C), with no further enhancement by CpG-ODN alone or with RA. Neither irradiation nor TLR9 stimulation affected pATR levels (Fig 3C). We also noted no effects of TLR9 stimulation on mRNA levels of DDR-specific genes known to be regulated on the transcriptional level in response to DNA damage (*ATM*, *BRCA2*, *DDB1*, *OGG1*, *UNG*, *MSH2*, *ERCC1;* S2 Fig).

### Expression of γH2AX as a measure of DSBs

**Fig 4:**
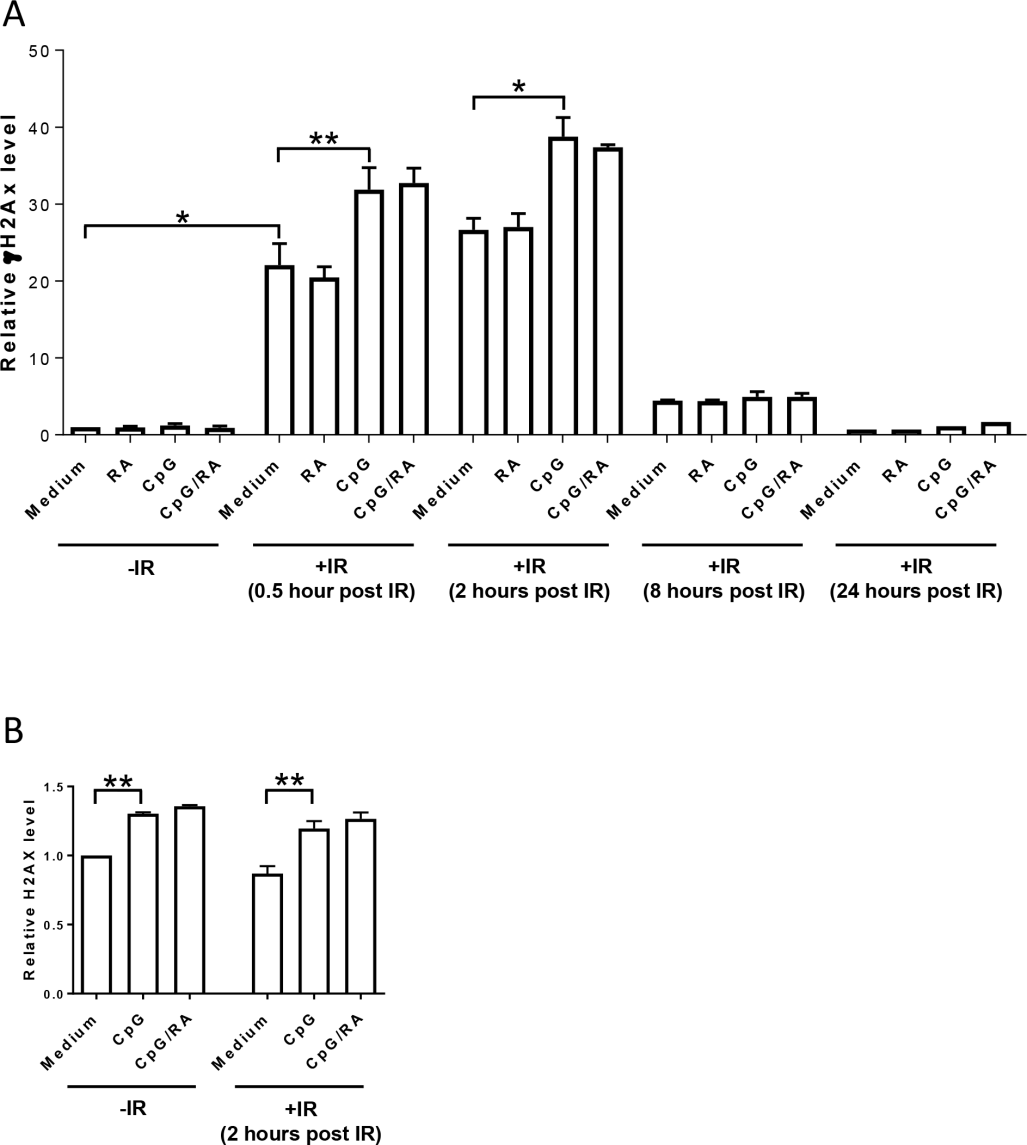
TLR9 stimulation enhances the levels of H2AX in B-cells. **(A)** B-cells were treated with CpG-ODN (0.5 μg/ml) in the presence or absence of RA (200 nM) for 24 hours prior to irradiation (IR; 10 Gy). After indicated time points, ethanol-fixed samples were bar-coded with pacific blue and four samples were mixed into a single tube before staining with antibodies recognizing γH2AX and DNA stain FxCycleTM Far Red. The median level of γH2AX was analyzed using flow cytometry. The histogram represents average of median levels of γH2AX ±SEM at indicated time points after irradiation related to non-irradiated cells (n = 3, *p < 0.05, **p < 0.01, paired t-test). **(B)** B-cells were treated as indicated in (A) and fixed with ethanol 2 hours after irradiation. The samples were barcoded and three samples were mixed in one tube before staining with antibodies recognizing H2AX and DNA stain FxCycleTM Far Red. The histograms present average of median levels of H2AX related to non-irradiated cells ±SEM (n = 4, *p < 0.05, **p < 0.01, paired t-test).

To assess induction of DSB as wells as DSB repair, we measured levels of γH2AX by phosphoflow analysis after irradiation of B-cells stimulated with CpG-ODN and RA [40;41]. We found that γH2AX levels increased in cells exposed to IR, with a maximum observed 2 hours after irradiation (Fig 4A). TLR9 stimulation enhanced the levels of γH2AX as measured 0.5 and 2 hours after irradiation, with no further enhancement imposed by RA. Similar effects on γH2AX levels measured 2 hours post-irradiation were noted by immunofluorescence microscopy (S3 Fig).

Based on the finding that TLR9-mediated stimulation of B-cells enhanced the expression of p53, we hypothesized that the elevated levels of γH2AX in response to TLR9 stimulation could be due to enhanced expression of H2AX rather than being a result of enhanced DNA damage. Indeed, our results showed that TLR9 stimulation of non-irradiated B-cells increases the levels of H2AX, with no additional effects of RA (Fig 4B). No further increase was noted upon irradiation, suggesting that TLR9-mediated enhancement of irradiation-induced γH2AX was merely due to CpG-ODN increasing the levels of total H2AX. Moreover, none of the stimulants affected the rate of DNA repair (Fig 4A).

### Measurement of strand breaks by comet assay

**Fig 5:**
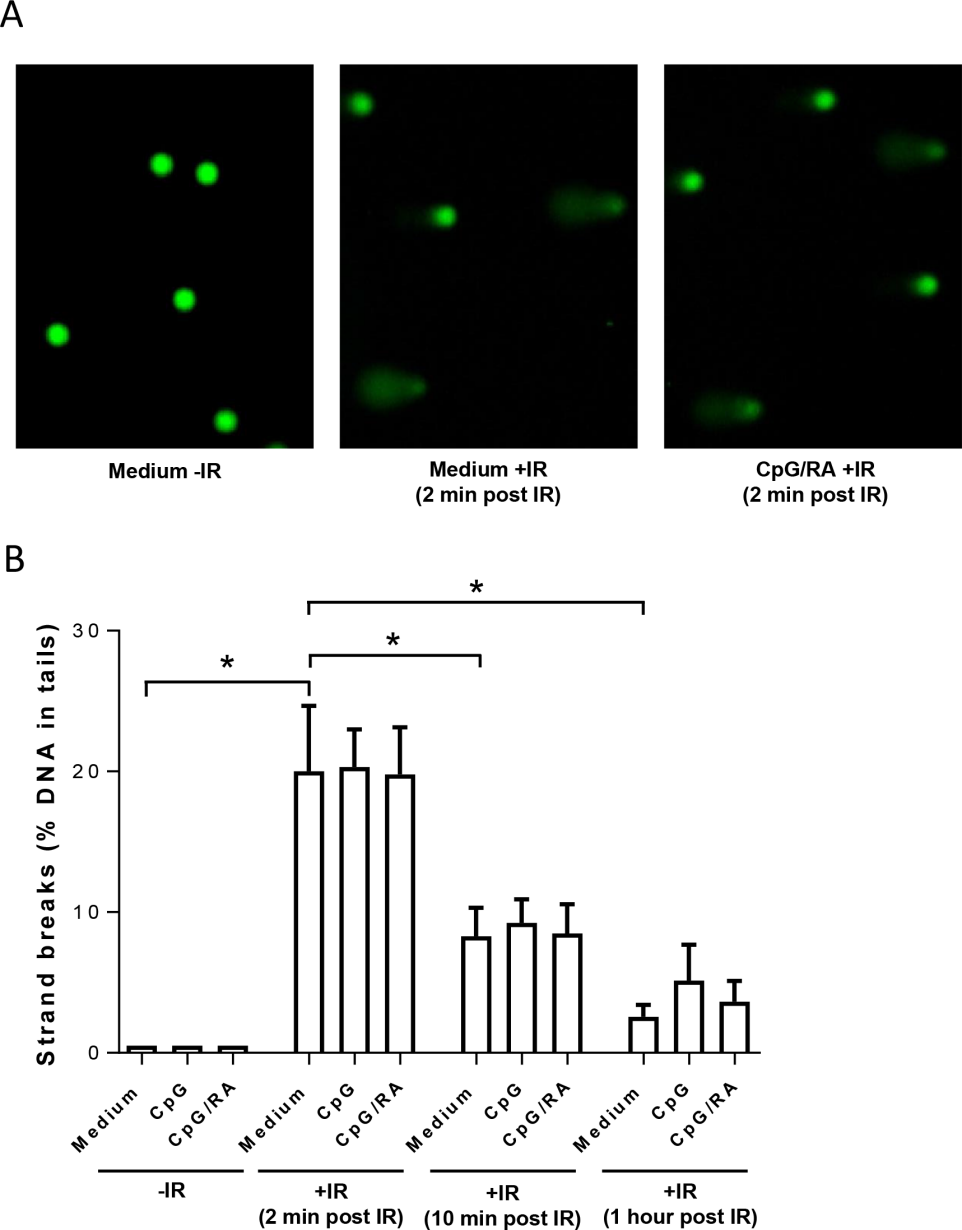
TLR9 stimulation does not enhance DNA damage-induced DNA strand breaks. B-cells were treated with CpG-ODN (0.5 μg/ml) in the presence or absence of RA (200 nM) for 24 hours prior to irradiation (IR; 5 Gy). After indicated time points the cells were lysed, and single cell gel electrophoresis was performed. DNA was stained with SYBRgold, and DNA damage was estimated measuring the % DNA in the tail. **(A)** Pictures of representative comets from non-irradiated and irradiated cells. One representative experiment is shown. **(B)** The results (% DNA in tails) are presented as histograms of mean values ±SEM (n = 4, *p < 0.05, paired t-test).

An alternative method to measure the induction of DNA damage is the alkaline comet assay. In this assay, DNA damage will induce relaxation of DNA supercoils allowing the DNA to migrate in an electric field, resulting in a “tail”. The alkaline comet assay enables the detection of both SSBs and DSBs in response to irradiation [34], and the gradual decline in strand breaks gives an estimation of the rate of DNA repair [42]. We found that irradiation immediately (within 2 minutes) increased the proportion of DNA in the comet tail, with no further enhancement imposed by CpG-ODN alone or with RA (Fig 5A and 5B). Furthermore, the DNA damage appeared to be rapidly repaired both in unstimulated and stimulated cells, as shown by the decline in strand breaks (Fig 5B).

## Discussion

The present study addresses the concern that B-cells stimulated via TLR9 and RA may survive with enhanced levels of DNA damage. We have previously shown that stimulating normal human B-cells with CpG-ODN and RA protects the cells from spontaneous and irradiation-induced cell death by enhancing levels of the anti-apoptotic proteins MCL1, BCL-XL and BCL2 [8]. Undoubtedly, protecting B-cells from DNA damage-induced cell death is beneficial when it comes to limiting immune suppression associated with for instance cancer treatment. Such protection would be particularly favorable for CVID patients, since they 1) often have limited number of functional B-cells, 2) retain B-cells with increased radiosensitivity and enhanced vulnerability to apoptosis, and 3) are frequently exposed to diagnostic radiation exposure due to recurrent infections and other complications [13;14;16]. In accordance with previous studies [12;13;17;18], we here show that B-cells from CVID patients generally are more prone to undergo cell death as compared to normal B-cells, and that the cells are less protected from cell death when stimulated by CpG-ODN. Still, also the CVID-derived B-cells were significantly protected from irradiation-induced cell death when pretreated with CpG-ODN in the presence or absence of RA. Enhanced survival of B-cells exposed to DNA-damaging treatment may however be a double-edged sword both for healthy individuals and CVID patients, since it potentially can increase the risk of mutations leading to B-cell malignancies.

To address this important issue, we measured the activation of the tumor suppressor p53, a key component in the DDR [35;36]. As expected, both the protein level and phosphorylation of p53 increased in response to irradiation. According to current hypothesis, p53 induces pro-apoptotic responses at levels above a certain threshold, whereas p53 levels below this threshold induce pro-survival responses [43]. In light of the reduced irradiation-mediated cell death imposed by TLR9 stimulation of B-cells, we expected that TLR9 stimulation also would reduce irradiation-induced p53 phosphorylation. It therefore came as a surprise that activation of TLR9 by CpG-ODN enhanced the irradiation-induced levels of phosphorylated p53, suggesting that TLR9 stimulation of B-cells augments irradiation-induced DNA damage. We were able to explain these contradictory results by demonstrating that TLR9 stimulation alone transcriptionally upregulates *TP53*, giving rise to enhanced levels of p53. This, in turn, results in elevated levels of phosphorylated p53 protein in irradiated cells, due to more p53 protein being available for phosphorylation. As irradiation alone did not induce transcription of TP53, these results support the notion that DNA damage mainly enhances the level of active p53 by promoting phosphorylation and stabilization of available protein, and not by enhanced *TP53* transcription [38]. Furthermore, the ability of TLR9 stimulation to transcriptionally upregulate *TP53* is in accordance with early studies from our own group [44] and others [45], showing that mitogenic stimulation of lymphocytes may result in increased transcription of p53. It has been proposed to be advantageous for cells to express low levels of p53, as this stimulates the cellular antioxidant defense [46;47]. Thus, the physiological relevance of increasing the levels of p53 in response to B-cell stimulation in non-irradiated cells, could be to induce a barrier against the risk of harboring ROS-mediated DNA damage that comes with the high proliferative rate in stimulated B-cells [37]. This TLR9-mediated effect on p53 could be of particular relevance in CVID patients, characterized by increased ROS production and disturbed glutathione metabolism [10;11]. In the absence of for example ROS-induced DNA damage, p53 would remain at low levels in an inactive state. In support of such a model, we did not observe phosphorylated p53 and expression of the p53-target gene *P21*^*Cip*^ in non-irradiated normal B-cells.

As a further verification of the assumption that stimulation of B-cells does not enhance irradiation-mediated DNA damage, we showed that neither TLR9 stimulation alone nor in the presence of RA further increased irradiation-induced strand breaks or phosphorylation of ATM and DNA-PKcs. The apparent enhancement of irradiation-induced γH2AX in stimulated cells was explained by the augmenting effects of CpG-ODN on the level of total H2AX in both irradiated and non-irradiated cells. In line with these results, it has been reported that factors unrelated to DNA damage may affect the levels of γH2AX. Hence, the level of H2AX doubles during the cell cycle [48] and the phosphorylation of H2AX is increased during mitosis unrelated to DNA damage [49]. The TLR9-mediated induction of H2AX was particularly interesting in light of the enhanced transcription of p53, implying that analyses of both γH2AX and phosphorylated p53 should be interpreted with caution when studying stimulated lymphocytes. The measured decline in γH2AX and DNA strand breaks suggested that DNA lesions induced by irradiation of B-cells are efficiently repaired, and that none of the stimulants affected the rate of repair. It should be emphasized that we in the present study only have measured the impact of TLR9/RA-mediated stimulation on the level of DNA strand breaks. Thus, we cannot exclude the possibility that activation of B cells may induce other types of injuries to DNA, such as oxidative DNA damage. We attempted to measure the induction of the purine oxidation product 8-oxoguanine by using lesion-specific enzymes when running the comet assay, but we were not able to obtain conclusive results due to the levels of apoptotic cells.

Having here established that stimulation of B-cells does not change the irradiation-induced activation of the DDR or the levels of DNA strand breaks in any direction–positively or negatively, how can we then explain the enhanced survival of the stimulated cells? In light of our previous results [8], we believe that TLR9-mediated survival of B-cells is the result of induced expression of the anti-apoptotic proteins BCL2, BCL-XL and MCL1. Furthermore, we consider the enhanced survival imposed by RA to be explained by the selective induction of MCL1.

We have previously proposed that CVID patients might benefit from treatment with CpG-ODN and vitamin A supplementation [32;50]. It was therefore encouraging to find that RA not only enhances the survival of TLR9-stimulated normal B-cells but also of CVID-derived B-cells. As CVID is a highly heterogeneous disease, one might of course question this conclusion based on the results from only 7 CVID patients. However, the effects of CpG-ODN and RA on the death of CVID B-cells followed the same trend in all 7 patients. Although both RA and CpG-ODN are considered as safe and are in clinical use for other purposes [51;52], we would ideally have liked to assess the effects of these compounds on the DDR and DNA strand breaks also in the patient-derived B-cells. We have, however, previously compared the levels of p53 mRNA in normal and CVID-derived B-cells in response to the combined stimulation via TLR9 and the Toll like receptor RP105, without finding any differences between the normal and diseased cells (S4 Fig).

Nevertheless, we believe that the main conclusion of the present study is important, i.e. that CpG-ODN/RA-stimulated B-cells survive without further enhancement of irradiation-induced DNA strand breaks. Furthermore, we suggest that the TLR9-mediated induction of inactive p53 is not due to enhanced DNA damage, but may rather be a “fit for fight” response developed to protect against ROS and other hazardous factors associated with enhanced cell proliferation. Our results support future use of CpG-ODN and vitamin A supplementation in treatment of CVID patients. Such treatment may not only improve deficient B-cell functions like proliferation and immunoglobulin production [32;50], but may also protect patients from DNA damage-imposed death of their vulnerable B-cells. It is conceivable that such effects will not be restricted to irradiation-induced DNA damage, but may also provide protection against other genotoxic mediators like enhanced oxidative stress.

## Supporting information

S1 Fig *TP53* levels in TLR9/RA stimulated B-cells. B-cells were stimulated with CpG-ODN (0.5 μg/ml) in the presence or absence of RA (200 nM) for 24 hours prior to isolation of mRNA. The level of *TP53* mRNA was quantified using RT-qPCR. The amount of *TP53* mRNA was related to the reference gene (*TBP*) and quantified using the 2^-ΔCt^-method. The results are presented as histograms of mean values ±SEM (n = 6 *p < 0.05, paired t-test).
(TIF)

S2 Fig mRNA expression of DDR-specific genes. B-cells were stimulated with CpG-ODNs (0.5 μg/ml) in the presence or absence of RA (200 nM) for 24 hours prior to irradiation (IR; 10 Gy). After additional 8 hours, the cells were harvested and subjected to RT-qPCR. The mRNA levels of target proteins were related to the reference gene (*TBP*) and quantified using the 2^-ΔCt^-method. The results are presented as histograms of one representative experiment.
(TIF)

S3 Fig γH2AX expression in TLR9/RA stimulated B-cells. B-cells were stimulated with CpG-ODNs (0.5 μg/ml) in the presence or absence of RA (200 nM) for 24 hours prior to irradiation (IR; 10 Gy). 2 hour after irradiation, the cells were subjected to immunofluorescence analysis as described in materials and methods. The results are presented as histograms of the intensity of γH2AX staining of 30 cells.
(TIF)

S4 Fig *TP53* levels in CVID-derived B-cells. Normal and CVID-derived B-cells were stimulated with CpG-ODNs (1 μg/ml) and anti-RP105 (1 μg/ml) for 72 hours prior to isolation of mRNA. The level of *TP53* mRNA was quantified using RT-qPCR, and the amount of *TP53* mRNA was related to the reference genes (TBP, B2M and 18s rRNA). The data represents mean 2^-ΔCt^ values ±SEM (n = 8).
(TIF)

S5 Fig Original uncropped Western blot of the expression of p53/p-p53. Original uncropped and unadjusted Western blot showing the level of p53 and p-p53 in Fig 2A.
(TIF)

S6 Fig Original uncropped Western blot of the expression of p21. Original uncropped and unadjusted Western blot blot showing the level of p21 in Fig 2C.
(TIF)

S7 Fig Original uncropped Western blot of pATM. Original uncropped and unadjusted Western blot showing the level of pATM in Fig 3A.
(TIF)

S8 Fig Original uncropped Western blot of pDNA-PKcs/pATR. Original uncropped and unadjusted Western blot showing the levels of pDNA-PKcs (upper panel) and pATR (lower panel) in Fig 3C.
(TIF)

S1 Raw data Raw data. Raw data showing the individual data points behind the means, medians and variances presented in the results, tables and figures in the manuscript.
(DOC)

S1 Table Characteristics of the CVID patients. The table presents sex, age and clinical manifestations of the CVID patients included in the study.
(DOC)
