# Supplementary figures and images for: TLR9 stimulation of B-cells induces transcription of p53 and prevents spontaneous and irradiation-induced cell death independent of DNA damage responses. Implications for Common variable immunodeficiency
Source: PLoS One. 2017 Oct 3;12(10):e0185708. doi: 10.1371/journal.pone.0185708 (PMC5626471; doi:10.1371/journal.pone.0185708)

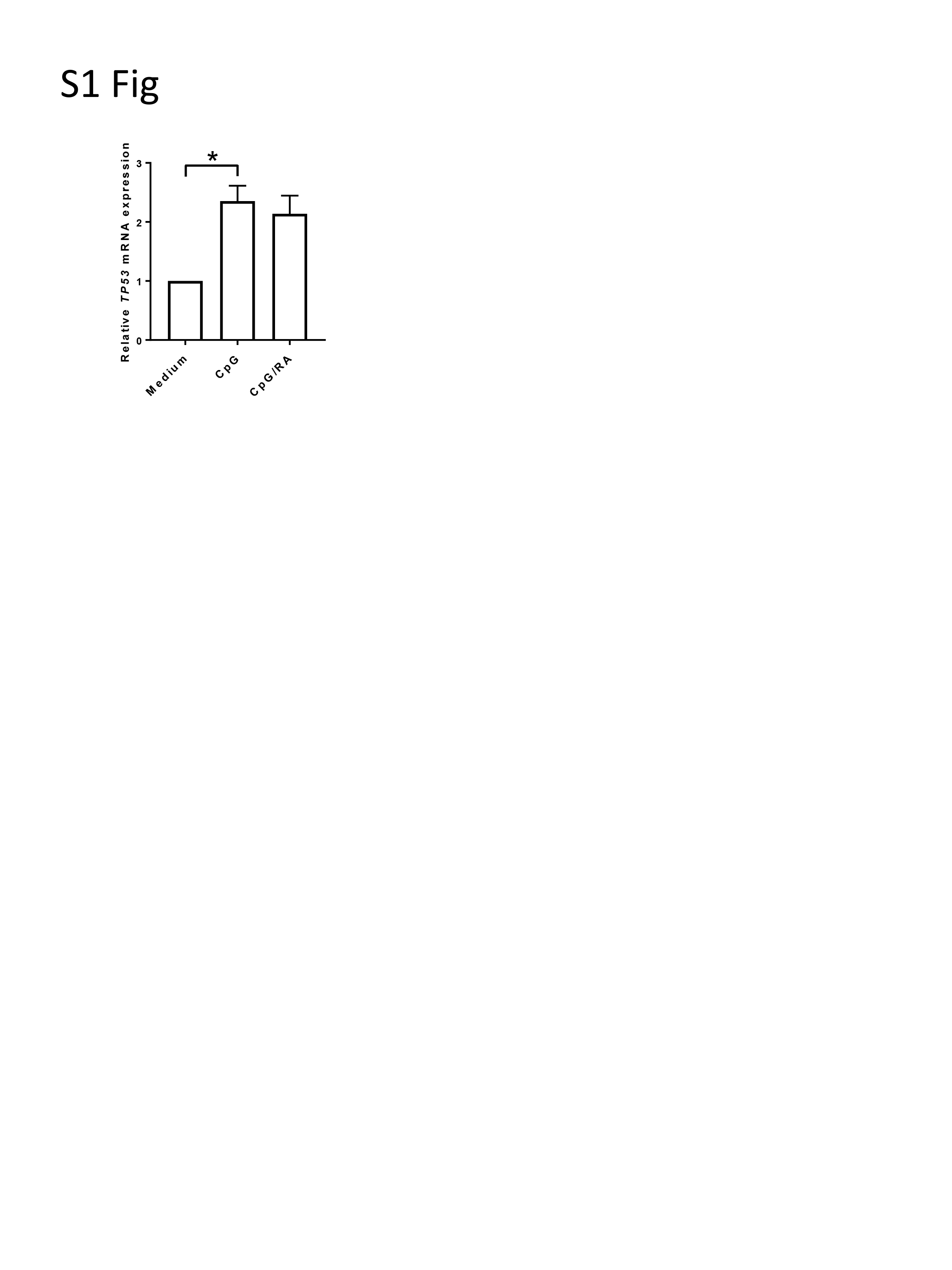

Supplement: S1 Fig — B-cells were stimulated with CpG-ODN (0.5 μg/ml) in the presence or absence of RA (200 nM) for 24 hours prior to isolation of mRNA. The level of TP53 mRNA was quantified using RT-qPCR. The amount of TP53 mRNA was related to the reference gene (TBP) and quantified using the 2-ΔCt-method. The results are presented as histograms of mean values ±SEM (n = 6 *p < 0.05, paired t-test). (TIF) [file pone.0185708.s001.tif]

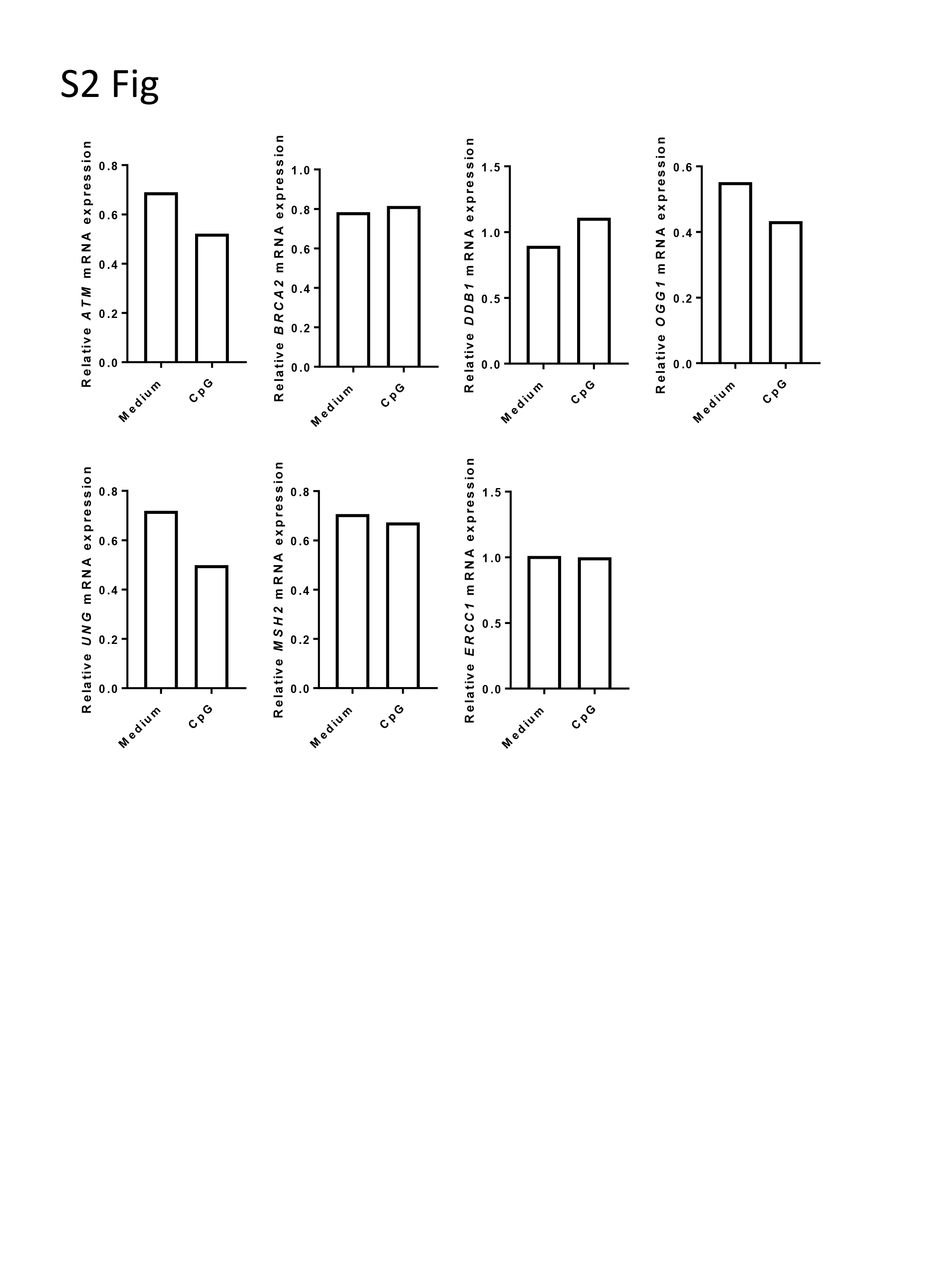

Supplement: S2 Fig — B-cells were stimulated with CpG-ODNs (0.5 μg/ml) in the presence or absence of RA (200 nM) for 24 hours prior to irradiation (IR; 10 Gy). After additional 8 hours, the cells were harvested and subjected to RT-qPCR. The mRNA levels of target proteins were related to the reference gene (TBP) and quantified using the 2-ΔCt-method. The results are presented as histograms of one representative experiment. (TIF) [file pone.0185708.s002.tif]

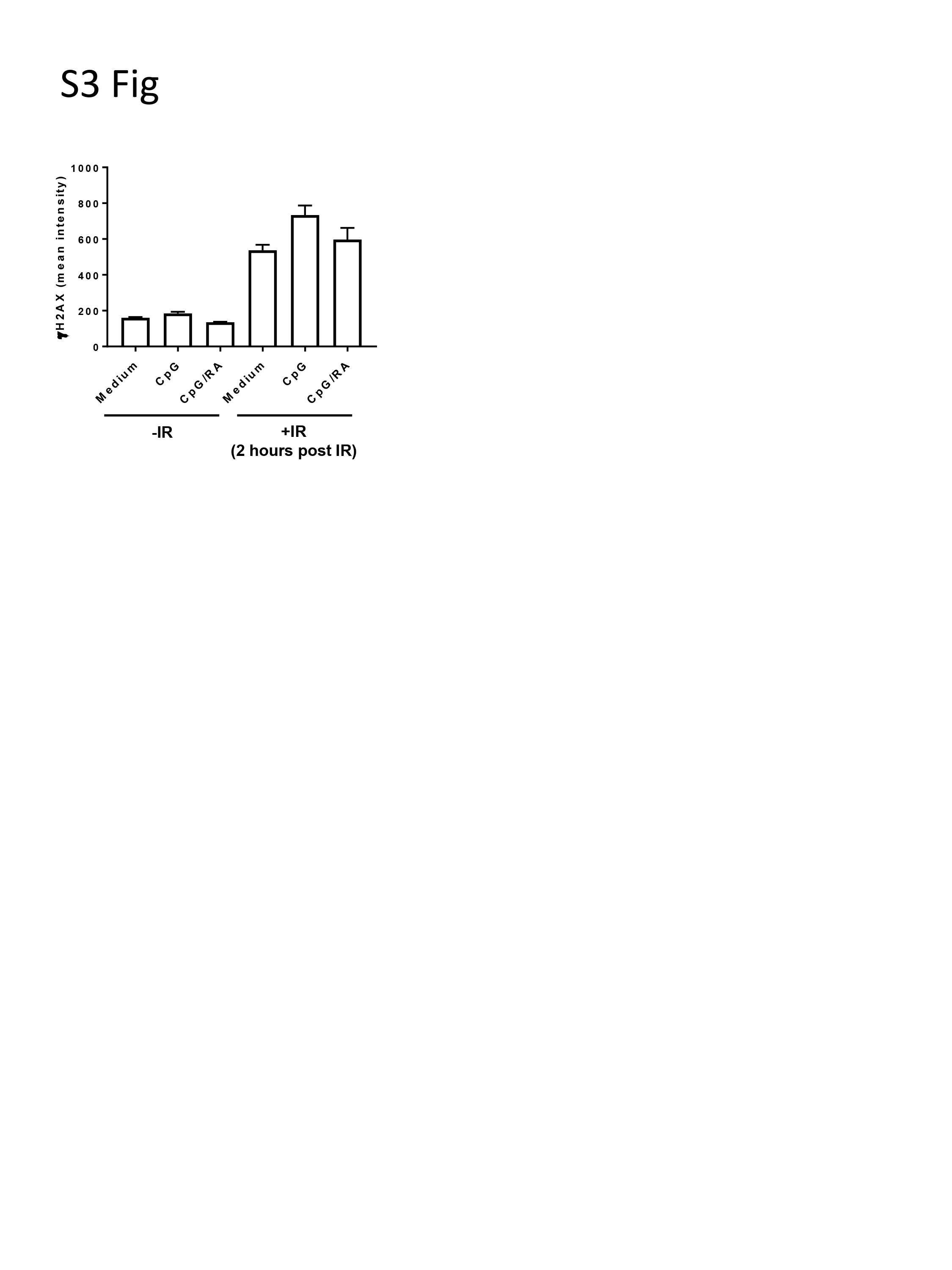

Supplement: S3 Fig — B-cells were stimulated with CpG-ODNs (0.5 μg/ml) in the presence or absence of RA (200 nM) for 24 hours prior to irradiation (IR; 10 Gy). 2 hour after irradiation, the cells were subjected to immunofluorescence analysis as described in materials and methods. The results are presented as histograms of the intensity of γH2AX staining of 30 cells. (TIF) [file pone.0185708.s003.tif]

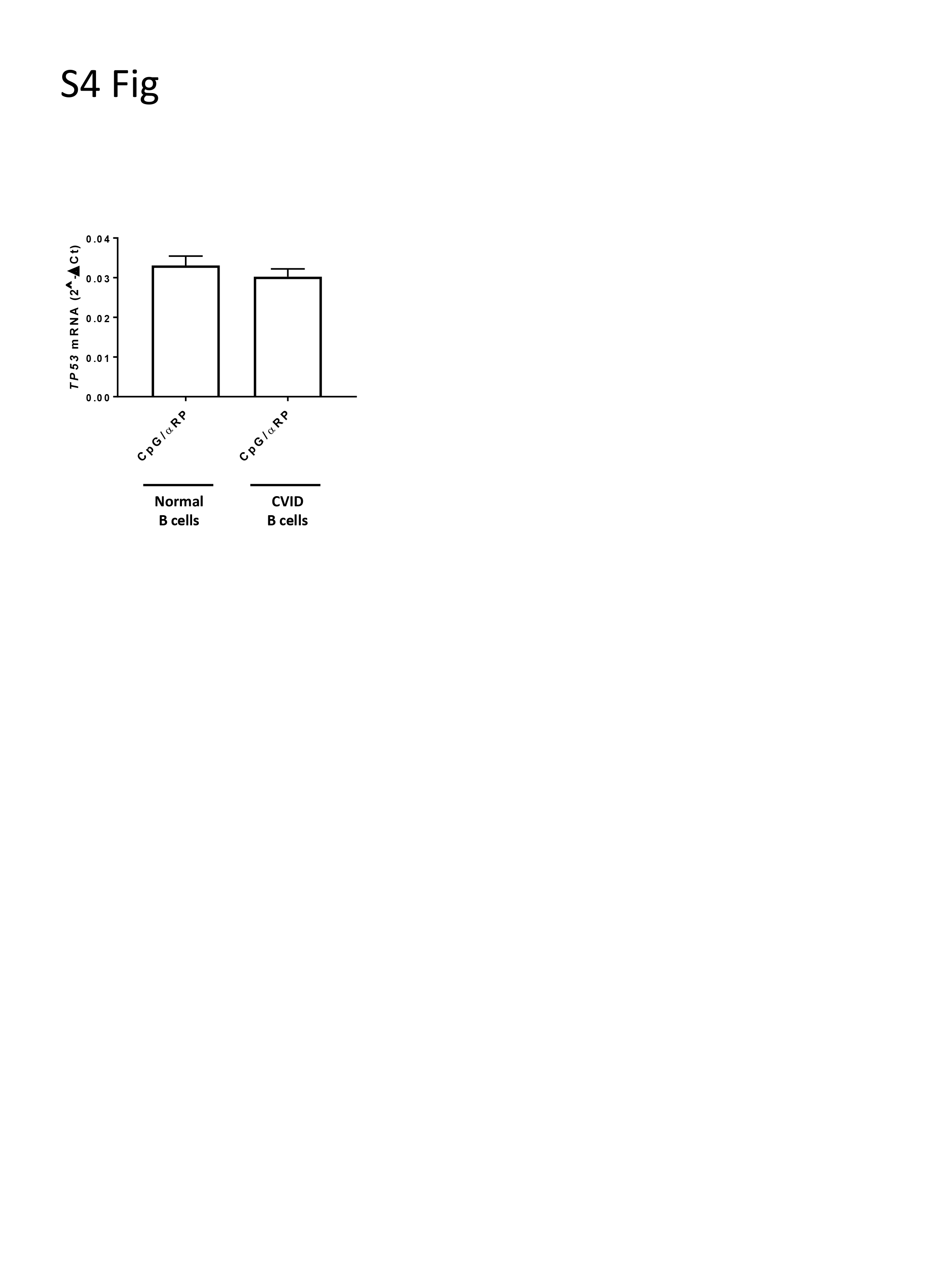

Supplement: S4 Fig — Normal and CVID-derived B-cells were stimulated with CpG-ODNs (1 μg/ml) and anti-RP105 (1 μg/ml) for 72 hours prior to isolation of mRNA. The level of TP53 mRNA was quantified using RT-qPCR, and the amount of TP53 mRNA was related to the reference genes (TBP, B2M and 18s rRNA). The data represents mean 2-ΔCt values ±SEM (n = 8). (TIF) [file pone.0185708.s004.tif]

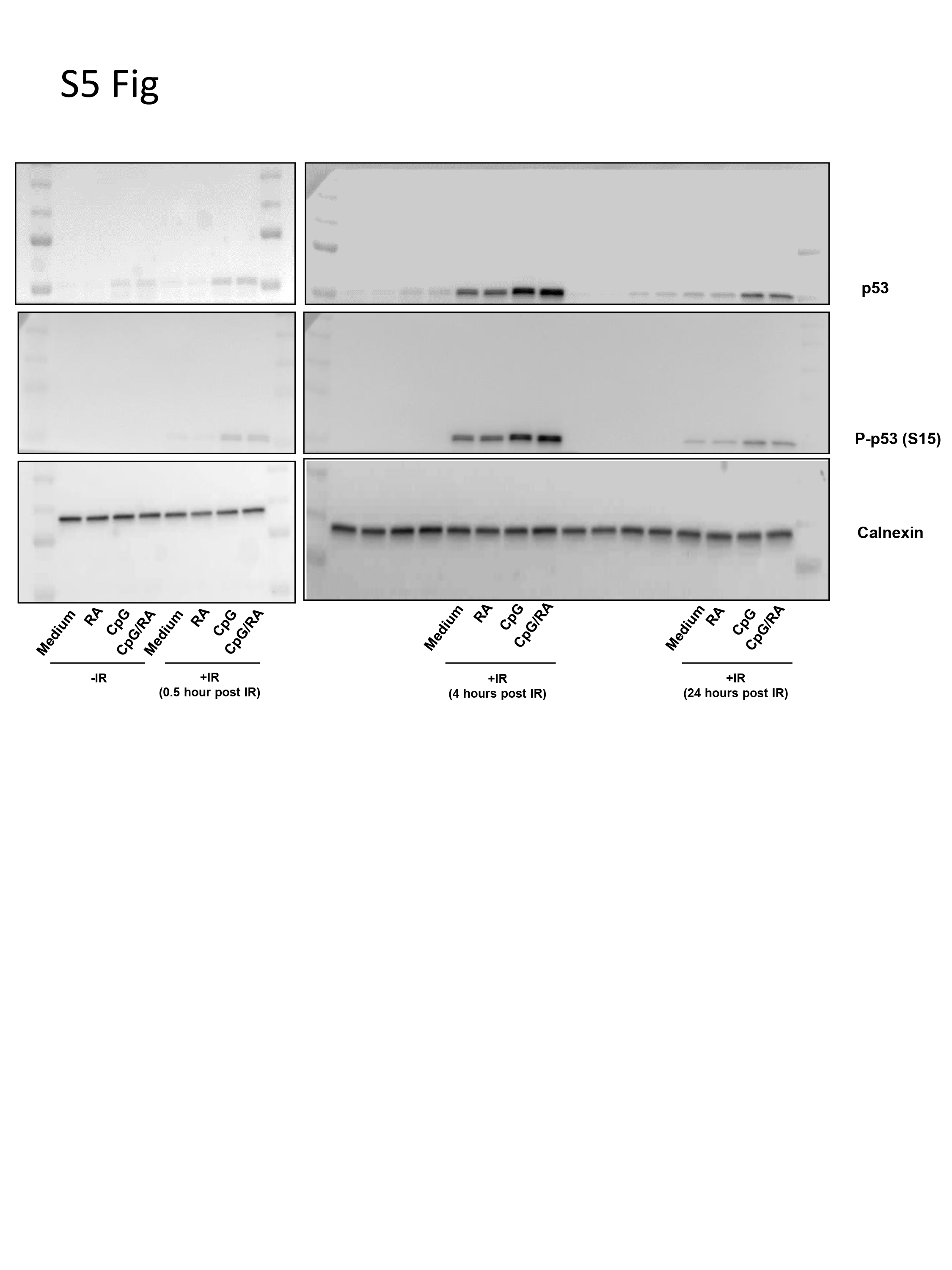

Supplement: S5 Fig — Original uncropped and unadjusted Western blot showing the level of p53 and p-p53 in Fig 2A. (TIF) [file pone.0185708.s005.tif]

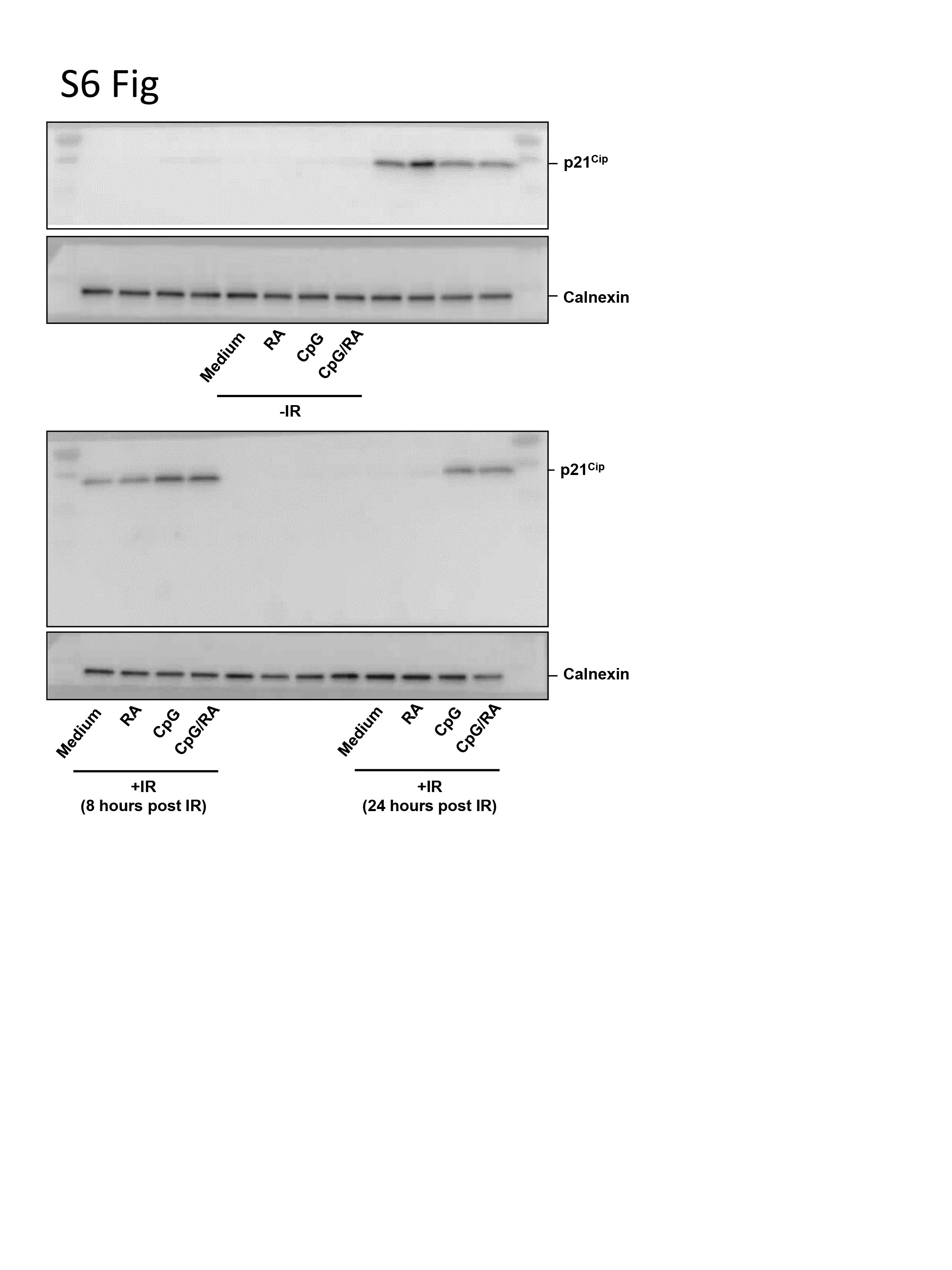

Supplement: S6 Fig — Original uncropped and unadjusted Western blot blot showing the level of p21 in Fig 2C. (TIF) [file pone.0185708.s006.tif]

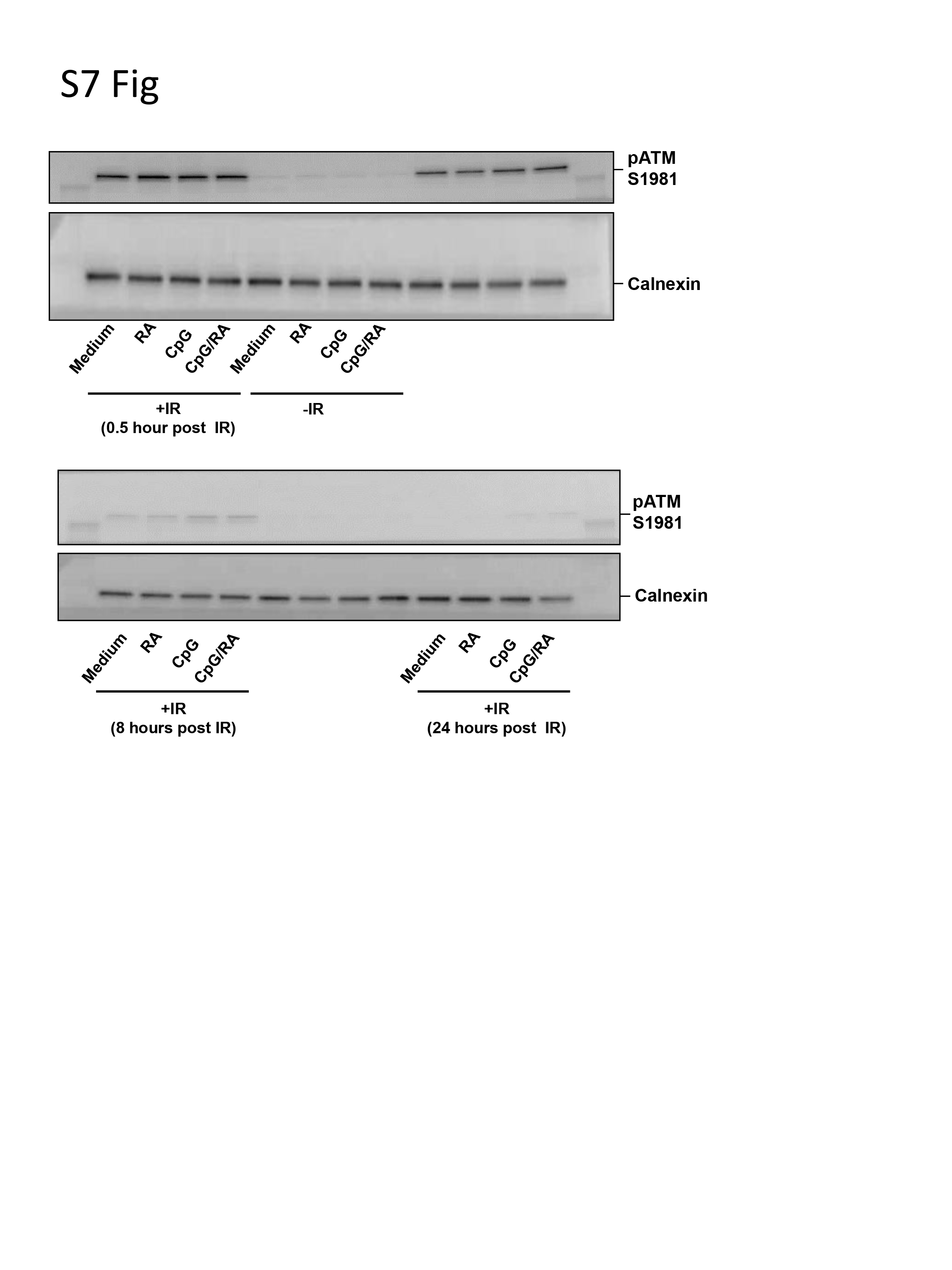

Supplement: S7 Fig — Original uncropped and unadjusted Western blot showing the level of pATM in Fig 3A. (TIF) [file pone.0185708.s007.tif]

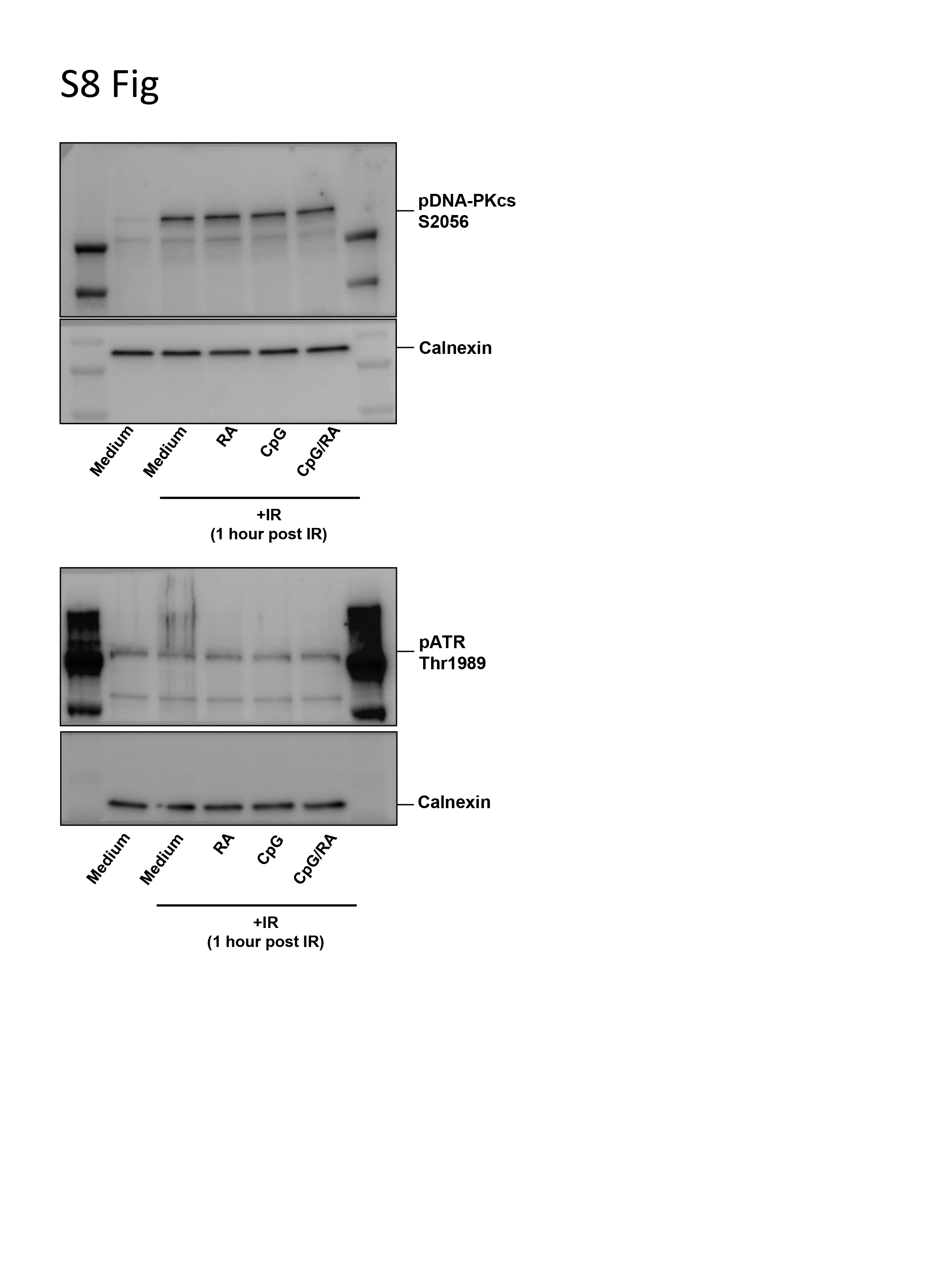

Supplement: S8 Fig — Original uncropped and unadjusted Western blot showing the levels of pDNA-PKcs (upper panel) and pATR (lower panel) in Fig 3C. (TIF) [file pone.0185708.s008.tif]
