# Supplementary material for: TLR9 stimulation of B-cells induces transcription of p53 and prevents spontaneous and irradiation-induced cell death independent of DNA damage responses. Implications for Common variable immunodeficiency
Source: PLoS One. 2017 Oct 3;12(10):e0185708. doi: 10.1371/journal.pone.0185708 (PMC5626471; doi:10.1371/journal.pone.0185708)
Supplement: S1 Raw data — Raw data showing the individual data points behind the means, medians and variances presented in the results, tables and figures in the manuscript. (DOC) [file pone.0185708.s009.doc]

| **Figure 1: Cell death measure by labeling cells with propidium iodide** | | | | | | | | |  |  |  |  |  |  |  |
| --- | --- | --- | --- | --- | --- | --- | --- | --- | --- | --- | --- | --- | --- | --- | --- |
| **Healthy donors** | | | | | | | | **CVID** | | | | | | | |
| **Non-irradiated** | | | | **Irradiated** | | | | **Non-irradiated** | | | | **Irradiated** | | | |
| **Medium** | **RA** | **CpG** | **CpG/RA** | **Medium** | **RA** | **CpG** | **CpG/RA** | **Medium** | **RA** | **CpG** | **CpG/RA** | **Medium** | **RA** | **CpG** | **CpG/RA** |
| 33,6 | 22,2 | 19,9 | 15,4 | 60,2 | 62,5 | 38,2 | 28,4 | 61,3 | 52,3 | 48,1 | 36,6 | 79,7 | 77,8 | 63,9 | 50,1 |
| 52,3 | 40,4 | 39,0 |  | 68,6 | 69,5 | 55,2 | 45,4 | 57,6 | 47,5 | 48,3 | 38,6 | 87,1 | 86,1 | 78,5 | 70,4 |
| 36,6 | 27,9 | 26,9 | 20,5 | 73,9 | 71,8 | 44,4 | 37,5 | 32,5 | 28,2 | 29,0 | 26,7 | 62,0 | 63,4 | 51,4 | 46,5 |
| 36,6 | 35,2 | 30,5 | 20,5 | 74,6 | 71,4 | 52,9 | 44,9 | 68,5 | 54,6 | 55,4 | 48,0 | 85,1 | 82,9 | 70,9 | 62,1 |
| 45,8 | 38,4 | 31,6 | 28,1 | 73,6 | 74,5 | 51,8 | 45,5 | 50,7 | 45,9 | 29,8 | 25,7 | 66,7 | 63,9 | 34,0 | 26,0 |
| 41,2 | 27,0 | 25,9 | 20,9 | 59,5 | 56,1 | 43,5 | 38,6 | 65,1 |  |  |  | 72,2 |  | 61,2 | 55,9 |
| 25,0 |  | 18,4 | 14,7 | 53,6 | 51,5 | 33,7 | 27,5 | 79,8 | 74,4 | 77,9 | 60,6 | 96,5 | 94,5 | 92,6 | 85,2 |

| **Figure 2 B: Relative p53 protein expression** | | | | |  |  |  |
| --- | --- | --- | --- | --- | --- | --- | --- |
| **Non-irradiated** | | | | **Irradiated** | | | |
| **Medium** | **RA** | **CpG** | **CpG/RA** | **Medium** | **RA** | **CpG** | **CpG/RA** |
| 1 | 1,18259 | 2,08163 | 2,30686 | 9,88428 | 11,484 | 18,0429 | 17,9129 |
| 1 |  |  |  | 11,8994 | 11,7176 | 12,1832 | 12,5599 |
| 1 | 1,02284 | 3,15808 | 4,71969 | 12,0737 | 14,8985 | 22,4204 | 17,6277 |
| 1 | 1,16501 | 3,34865 | 4,41058 | 22,0413 | 23,0993 | 37,5389 | 35,2683 |
| 1 | 1,03774 | 1,69411 | 1,79739 | 6,42245 | 7,1249 | 9,8492 | 13,2061 |
| 1 |  |  |  | 7,9321 | 8,10765 | 11,4143 | 10,9868 |

| **Figure 2 D: Relative *TP53* mRNA expression** | | | | |  |
| --- | --- | --- | --- | --- | --- |
| **Non-irradiated** | | | **Irradiated** | | |
| **Medium** | **CpG** | **CpG/RA** | **Medium** | **CpG** | **CpG/RA** |
| 1 | 3,18131 | 3,93277 | 0,949 | 2,74879 | 3,61205 |
| 1 | 3,19299 | 2,70977 | 1,15604 | 1,76162 | 3,09143 |
| 1 | 1,69042 | 2,7511 | 1,24291 | 2,25359 | 2,63804 |
| 1 | 2,02455 | 2,36155 | 0,96357 | 2,33215 | 2,12784 |

| **Figure 3 B: Relative pATM expression** | | | | |  |  |  |
| --- | --- | --- | --- | --- | --- | --- | --- |
| **Non-irradiated** | | | | **Irradiated** | | | |
| **Medium** | **RA** | **CpG** | **CpG/RA** | **Medium** | **RA** | **CpG** | **CpG/RA** |
| 1 |  |  |  | 3,49515 | 4,091 | 3,39021 | 3,38022 |
| 1 |  |  |  | 3,44749 | 4,55136 | 3,61368 | 2,89944 |
| 1 | 1,3023 | 0,9812 | 1,33563 | 9,44974 | 9,60783 | 7,53431 | 7,53373 |
| 1 | 0,7599 | 0,74515 | 0,5933 | 4,92655 | 4,03081 | 5,44866 | 4,52412 |
| 1 |  |  |  | 5,71976 | 6,01935 | 5,3286 | 5,68972 |

| **Figure 4 A: Relative γH2AX level** | | | | |  |  |  |  |  |  |  |  |  |  |  |  |  |  |  |
| --- | --- | --- | --- | --- | --- | --- | --- | --- | --- | --- | --- | --- | --- | --- | --- | --- | --- | --- | --- |
| **Non-irradiated** | | | | **Irradiated 0.5 hour post IR** | | | | **Irradiated 2 hours post IR** | | | | **Irradiated 8 hours post IR** | | | | **Irradiated 24 hours post IR** | | | |
| **Medium** | **RA** | **CpG** | **CpG/RA** | **Medium** | **RA** | **CpG** | **CpG/RA** | **Medium** | **RA** | **CpG** | **CpG/RA** | **Medium** | **RA** | **CpG** | **CpG/RA** | **Medium** | **RA** | **CpG** | **CpG/RA** |
| 1,0 |  | 0,9 | 0,7 | 27,3 | 23,3 | 36,9 | 36,1 | 29,4 | 29,3 | 41,7 | 37,4 | 4,5 | 4,1 | 5,5 | 4,3 |  |  |  |  |
| 1,0 | 0,8 | 1,0 | 0,6 | 21,3 | 19,1 | 31,7 | 32,6 | 24,3 | 23,5 | 33,8 | 36,8 | 4,2 | 4,3 | 3,6 | 4,7 | 0,7 | 0,7 | 1,1 | 1,7 |
| 1,0 | 1,1 | 1,7 | 1,4 | 17,6 | 19,0 | 27,0 | 29,5 | 26,2 | 28,2 | 40,8 | 38,0 | 4,5 | 4,7 | 5,7 | 5,9 |  |  |  |  |

| **Figure 4 B: Relative H2AX level** | | | |  |  |
| --- | --- | --- | --- | --- | --- |
| **Non-irradiated** | | | **Irradiated** | | |
| **Medium** | **CpG** | **CpG/RA** | **Medium** | **CpG** | **CpG/RA** |
| 1 | 1,284422 | 1,348544 | 1,00236 | 1,24705 | 1,372148 |
| 1 | 1,283637 | 1,371166 | 0,910301 | 1,314815 | 1,287254 |
| 1 | 1,322936 | 1,372967 | 0,770169 | 1,148609 | 1,257192 |
| 1 | 1,319862 | 1,332518 | 0,79244 | 1,073405 | 1,143436 |

| **Figure 5 B: Strand breaks (% DNA in tail)** | | | | |  |  |  |  |  |  |  |
| --- | --- | --- | --- | --- | --- | --- | --- | --- | --- | --- | --- |
| **Non-irradiated** | | | **Irradiated 2 min post IR** | | | **Irradiated 10 min post IR** | | | **Irradiated 1 hour post IR** | | |
| **Medium** | **CpG** | **CpG/RA** | **Medium** | **CpG** | **CpG/RA** | **Medium** | **CpG** | **CpG/RA** | **Medium** | **CpG** | **CpG/RA** |
| 0 | 0 | 0 | 13,8 | 13,45 | 13,35 |  |  |  | 0 | 0,37 | 0,45 |
| 0 | 0 | 0 | 10,3 | 18,8 | 14,95 | 5,2 | 7,25 | 5,35 | 3,65 |  | 1,925 |
| 0 | 0 | 0 | 28,45 | 24,425 | 23,775 | 12,075 | 12,575 | 12,4 | 3,2 | 9,05 | 6,95 |
| 0 | 0 | 0 | 27,5 | 24,6 | 27,1 | 7,6 | 7,9 | 7,7 | 3,4 | 6 | 5,2 |

| **S1 Fig: relative TP53 mRNA expression** | | |
| --- | --- | --- |
| **Medium** | **CpG** | **CpG/RA** |
| 1 | 1,45 | 1,23 |
| 1 | 2,6 | 1,43 |
| 1 | 2,02 | 2,36 |
| 1 | 3,13 | 2,45 |
| 1 | 2,01 | 2,06 |
| 1 | 2,91 | 3,3 |

| **S2 Fig: mRNA expression of DDR-specific genes** | | | |
| --- | --- | --- | --- |
|  | **Non-irradiated** | **Irradiated** | |
|  | **Medium** | **Medium** | **CpG** |
| **ATM** | 1 | 0,69 | 0,522 |
| **BRCA2** | 1 | 0,784 | 0,815 |
| **DDB1** | 1 | 0,896 | 1,11 |
| **OGG1** | 1 | 0,552 | 0,433 |
| **UNG** | 1 | 0,719 | 0,499 |
| **MHS2** | 1 | 0,707 | 0,673 |
| **ERCC1** | 1 | 1,01 | 1 |

| **S3 Fig: γH2AX expression (mean intensity)** | | | |  |  |
| --- | --- | --- | --- | --- | --- |
| **Non-irradiated** | | | **Irradiated** | | |
| **Medium** | **CpG** | **CpG/RA** | **Medium** | **CpG** | **CpG/RA** |
| 159,04 | 163,53 | 121,63 | 959,90 | 449,80 | 447,88 |
| 159,24 | 155,04 | 123,12 | 519,23 | 362,83 | 366,40 |
| 170,26 | 138,94 | 131,38 | 873,59 | 302,66 | 462,92 |
| 144,96 | 146,12 | 126,31 | 782,52 | 762,01 | 451,03 |
| 147,29 | 138,88 | 171,96 | 442,92 | 546,87 | 395,77 |
| 130,59 | 140,01 | 125,64 | 741,42 | 664,66 | 524,88 |
| 128,78 | 140,76 | 130,78 | 347,19 | 587,05 | 454,58 |
| 162,97 | 143,56 | 130,15 | 563,84 | 657,91 | 532,83 |
| 169,24 | 185,41 | 121,37 | 389,85 | 1076,08 | 788,61 |
| 147,14 | 155,56 | 132,72 | 488,80 | 620,76 | 394,62 |
| 135,24 | 154,42 | 129,71 | 448,93 | 747,27 | 473,76 |
| 139,33 | 156,70 | 128,37 | 318,92 | 1588,06 | 475,08 |
| 138,93 | 140,79 | 133,19 | 645,02 | 1352,91 | 308,72 |
| 145,69 | 195,24 | 128,14 | 338,79 | 665,38 | 555,51 |
| 148,07 | 191,45 | 127,27 | 373,16 | 586,94 | 446,02 |
| 151,35 | 168,68 | 138,01 | 515,91 | 730,18 | 455,52 |
| 153,97 | 169,45 | 130,71 | 523,91 | 651,03 | 329,20 |
| 161,09 | 156,82 | 140,82 | 425,41 | 757,57 | 421,71 |
| 152,41 | 179,96 | 130,45 | 712,73 | 1157,11 | 657,63 |
| 158,57 | 231,23 | 133,88 | 512,29 | 1018,38 | 1476,21 |
| 164,67 | 197,90 | 137,53 | 538,08 | 685,42 | 1579,49 |
| 190,23 | 163,46 | 133,81 | 426,26 | 595,33 | 903,22 |
| 226,17 | 188,89 | 193,26 | 662,10 | 1248,32 | 529,67 |
| 195,98 | 190,31 | 130,09 | 532,24 | 926,14 | 500,07 |
| 177,37 | 343,94 | 156,97 | 327,94 | 1020,96 | 994,58 |
| 153,82 | 182,63 | 150,36 | 499,60 | 514,45 |  |
| 143,17 | 220,22 | 124,28 | 784,21 | 782,49 |  |
| 167,07 | 276,98 | 126,99 | 402,42 | 591,55 |  |
| 187,24 | 303,32 |  | 375,58 | 521,26 |  |
| 201,95 | 211,32 |  | 773,90 | 524,29 |  |
| 167,59 |  |  | 391,54 | 473,75 |  |
|  |  |  |  | 314,30 |  |

| **S4 Fig: *TP53* mRNA levels (2˄-ΔCt)** | |
| --- | --- |
| **Normal B cells** | **CVID B cells** |
| **CpG/αRP** | **CpG/αRP** |
| 0,036222 | 0,023422 |
| 0,026151 | 0,036046 |
| 0,025648 | 0,03125 |
| 0,045721 | 0,027167 |
| 0,034435 | 0,025489 |
| 0,035256 | 0,036651 |
| 0,034008 | 0,025365 |
| 0,027337 | 0,036499 |
