# Supplementary material for: TLR9 stimulation of B-cells induces transcription of p53 and prevents spontaneous and irradiation-induced cell death independent of DNA damage responses. Implications for Common variable immunodeficiency
Source: PLoS One. 2017 Oct 3;12(10):e0185708. doi: 10.1371/journal.pone.0185708 (PMC5626471; doi:10.1371/journal.pone.0185708)
Supplement: S1 Table — The table presents sex, age and clinical manifestations of the CVID patients included in the study. (DOC) [file pone.0185708.s010.doc]

| **ID** | **Gender** | **Age (years)** | **Clinical manifestations** |
| --- | --- | --- | --- |
| **1** | Female | 34 | Splenomegaly, immunologic thrombocytopenic purpura |
| **2** | Male | 80 | Psoriasis with arthropathy, sacroileitis |
| **3** | Male | 39 | Infection only |
| **4** | Female | 68 | Lymphadenopathy, splenomegaly |
| **5** | Female | 52 | Infection only |
| **6** | Female | 56 | Arthritis, splenomegaly |
| **7** | Female | 45 | Splenomegaly |
